# The Efficiency of Selected Extenders against Bacterial Contamination of Boar Semen in a Swine Breeding Facility in Western Slovakia

**DOI:** 10.3390/ani11113320

**Published:** 2021-11-20

**Authors:** Eva Tvrdá, Ondřej Bučko, Kristína Rojková, Michal Ďuračka, Simona Kunová, Ján Kováč, Filip Benko, Miroslava Kačániová

**Affiliations:** 1 Institute of Applied Biology, Faculty of Biotechnology and Food Sciences, Slovak University of Agriculture, Tr. A. Hlinku 2, 949 76 Nitra, Slovakia; kristinarojkova@icloud.com (K.R.); michaelduracka@gmail.com (M.Ď.); jan.johnny.kovac@gmail.com (J.K.); filip.benko276@gmail.com (F.B.); 2 Institute of Animal Husbandry, Faculty of Agrobiology and Food Resources, Slovak University of Agriculture, Tr. A. Hlinku 2, 949 76 Nitra, Slovakia; ondrej.bucko@uniag.sk; 3 Institute of Food Sciences, Faculty of Biotechnology and Food Sciences, Slovak University of Agriculture, Tr. A. Hlinku 2, 949 76 Nitra, Slovakia; simona.kunova@uniag.sk; 4 Institute of Horticulture, Faculty of Horticulture and Landscape Engineering, Slovak University of Agriculture, Tr. A. Hlinku 2, 949 76 Nitra, Slovakia; miroslava.kacaniova@gmail.com; 5 Department of Bioenergetics, Food Analysis and Microbiology, Institute of Food Technology and Nutrition, University of Rzeszow, Cwiklinskiej 1, 35-601 Rzeszow, Poland

**Keywords:** bacteriospermia, boars, semen extender, antibiotics, MALDI-TOF, bacterial resistance

## Abstract

**Simple Summary:**

This study evaluated the efficiency of selected semen extenders to prevent bacterial overgrowth in boar ejaculates stored for 72 h. Among the identified bacterial isolates, *Escherichia coli* and *Pseudomonas aeruginosa* were the most prevailing species. While all extenders supplemented with antibiotics ensured a satisfactory sperm vitality during the storage period, neither of them was able to achieve a complete elimination of bacteria from extended semen. Furthermore, a number of bacterial isolates exhibited resistance to several antibiotics chosen for the microbial susceptibility test (e.g., tigecyklin and ciprofloxacin).

**Abstract:**

Bacteriospermia has become a serious factor affecting sperm quality in swine breeding, this is why antibiotics (ATBs) are a critical component of semen extenders. Due to ever-increasing antimicrobial resistance, the aim of this study was to assess the efficiency of selected commercially available semen extenders to prevent a possible bacterial contamination of boar ejaculates. Three Androstar Plus extenders containing different combinations of antibiotics were used to process ejaculates from 30 healthy Duroc breeding boars. Androstar Plus without antibiotics was used as a control. The extended samples were stored at 17 °C for 72 h. Sperm motility, viability, mitochondrial activity, DNA integrity and oxidative profile of each extended sample were assessed following 24 h, 48 h and 72 h. Furthermore, selective media were used to quantify the bacterial load and specific bacterial species were identified with matrix-assisted laser desorption/ionization time-of-flight (MALDI-TOF) mass spectrometry. The results indicate that semen extenders enriched with ATBs ensured a significantly higher preservation of the sperm quality in comparison to the ATB-free control. The total bacterial count was significantly decreased in the extenders supplemented with ATBs (*p* < 0.001), however gentamycin alone was not effective enough against Gram-positive bacteria, while a few colonies of *Enterococcus hirae*, *Bacillus subtilis* and *Corynebacterium* spp. were present in the samples extended in the presence of a triple combination of ATBs. In conclusion, we may suggest that semen extenders enriched in antibiotics were not able to fully eliminate the bacteria present in the studied samples. Furthermore, selection of suitable antibiotics for semen extension should be accompanied by adequate hygiene standards during the collection and handling of boar ejaculates.

## 1. Introduction

The evolution of contemporary swine industry is closely associated with the implementation of modern reproductive technologies which have significantly contributed to a remarkable rise in the production of high-quality protein foodstuffs [1]. In particular, artificial insemination (AI) has become a popular choice for an intensive pig production, since approximately 90% of sows are being fertilized artificially in the leading pork producing countries [1,2]. In comparison to natural mating, AI is a more effective tool to accelerate the genetic progress of breeding, while minimizing the risks of horizontal or vertical transmission of venereal diseases [1,3]. Nonetheless, the success of AI directly depends on the quality of the semen sample used for the procedure.

Amongst numerous endogenous and exogenous factors that may contribute to a decreased fertility in animal breeding, bacterial contamination of semen may be also responsible for a reduced shelf life of extended semen, conception rates and litter size [2,4,5,6]. Bacteriospermia in boars may originate from the urogenital system or preputial fluids as well as hair, skin, respiratory secretions, or feces. Contaminated water and feed, air ventilation, bedding or poor hygiene conditions of the breeding facility may similarly lead to bacterial infestation of semen [7].

It has been previously reported that 43–100% of raw boar ejaculates contain bacteria [4,5,6]. What is more, approximately three quarters of extended semen specimens may present with bacterial contamination [2,4,5,8], which may have a negative impact on the success of artificial insemination in swine production. Although extenders for boar semen have become routine in pig breeding since the 1970s [9], boar spermatozoa are exceptionally prone to cold shock and oxidative stress, which is why temperatures oscillating around 15–20 °C are considered to be ideal for the storage of boar semen [10]. Nevertheless, room temperatures and media rich in nutrients favor the growth of bacteria with subsequent deleterious effects on the sperm survival [2,4,7,11].

Bacterial contamination of extended ejaculates has been previously associated with sperm agglutination [4,6], a reduced sperm motion activity [11,12], alterations to the membrane [3] and acrosome [13], rendering the affected semen sample to be less efficient in accomplishing fertilization. Furthermore, bacteria may accelerate reactive oxygen species (ROS) overproduction [14], leading to increased insults to the sperm lipids, proteins, and DNA [15].

To overcome complications associated with high bacterial load in AI doses, current legislation (Council Directive, European Union, 90/429/EEC) requests the addition of antibiotics to each extended semen sample. Meanwhile, continuous use of antibiotics supports the occurrence, spread and persistence of multidrug-resistant bacteria [16]. As a matter of fact, previous studies have unraveled resistance among isolates from boar ejaculates against antibiotics commonly used as supplements in commercial semen extenders, such as penicillin, streptomycin, and gentamycin [5,11].

As such, our objectives were to: (a) investigate the bacteriological profiles of extended boar semen using a molecular approach based on the matrix assisted laser desorption/ionization time-of-flight (MALDI-TOF) mass spectrometry; (b) to assess the sensitivity of isolated bacteria to antibiotics, and (c) to assess the ability of selected commercially available boar semen extenders supplemented with different antibiotics to eliminate bacteria during semen storage, and thus to offer protection to the sperm structural integrity and functional activity.

## 2. Materials and Methods

### 2.1. Semen Collection and Dilution

Four varieties of the Androstar Plus boar semen extender (Minitüb, Tiefenbach, Germany) were used for the study: Androstar Plus without antibiotics served as the control (Ctrl), while the experimental groups consisted of Androstar Plus containing gentamycin exclusively (Experimental group 1; Exp 1), Androstar Plus supplemented with gentamicin, aminoglycosid and cephalosporin (Experimental group 2; Exp 2) and Androstar Plus with gentamicin, lincomycin and spectinomycin (Experimental group 3; Exp 3). Two hours prior to the semen collection, all media were prepared according to the recommendations of the manufacturer.

Semen samples were acquired from 30 adult (2–3 years old) breeding Duroc boars housed at the pig farm Terezov (Hlohovec, Slovakia) during March 2020. Disposable gloves were changed between each animal. Prior to the collection, the boars were allowed to urinate, and their external genitalia were washed with soap and water [17,18].

Sperm-rich fractions were collected by a qualified technician using the gloved-hand technique, and subsequently transported to the andrology laboratory in an isothermal vessel (37 °C) within 45 min. Each ejaculate was subjected to a primary assessment of volume, sperm concentration and motility. The samples had to accomplish the given criteria (volume > 200 mL, concentration > 200 × 10^6^ sperm/mL, motility > 70%) for subsequent procedures involving semen dilution and storage [17].

Each semen sample was divided into 4 equal fractions and each part was diluted either with the control or experimental medium using a dilution ratio of 1:20. The diluted samples were stored under controlled temperature conditions (17 °C). Sperm quality and bacteriological assessments were performed immediately following dilution (0 h) as well as 24 h, 48 h, and 72 h post-dilution.

Prior to each round of analysis, 10 mL of each diluted semen was pre-warmed to 37 °C and placed into a sterile Class II laminar flow hood. An aliquot of each specimen was transferred into a sterile Eppendorf tube and stored at −20 °C for bacteriological analyses [15].

### 2.2. Sperm Motility

Sperm motility (MOT; %) was assessed with the HTM TOX IVOS II. Computer-assisted sperm analysis (CASA) system (Hamilton-Thorne Biosciences, Beverly, MA, USA). In order for the system to differentiate between spermatozoa and bacteria, the samples were stained using the IDENT stain (Hamilton-Thorne Biosciences, Beverly, MA, USA) and analyzed under fluorescent illumination. Each sample was loaded into the Makler counting chamber (Sefi Medical Instruments, Haifa, Israel) and a minimum of 1000 spermatozoa were evaluated [19].

### 2.3. Sperm Viability

To assess the sperm viability, 1 × 10^6^ cells were adjusted to 100 μL with PBS (Dulbecco’s phosphate buffered saline; Sigma-Aldrich, St. Louis, MO, USA), triple-stained with 10 μL CFDA (carboxyfluorescein diacetate; Sigma-Aldrich, St. Louis, MO, USA; 0.75 mg/mL in DMSO), 10 μL PI (propidium iodide; Sigma-Aldrich, St. Louis, MO, USA; 5 μg/mL in PBS) and 10 μL DAPI (4′,6-diamidino-2-phenylindole; Sigma-Aldrich, St. Louis, MO, USA; 1 μM in PBS) and incubated for 15 min at 37 °C in the dark. Subsequently, the samples were centrifuged for 5 min at 300× *g* and washed with 100 µL PBS twice. Finally, the cells were resuspended in 100 µL PBS, and at least 300 spermatozoa were examined under an epifluorescence microscope with a 40× magnification objective (Leica Microsystems, Wetzlar, Germany). Spermatozoa exhibiting CFDA-positivity were considered to be membrane-intact (%) while PI-positive cells were classified as necrotic (%) [17].

### 2.4. Acrosome Integrity

In case of the acrosome integrity, 1 × 10^6^ cells were diluted to 100 μL with PBS, and stained with 100 μL PNA (peanut agglutinin, FITC conjugate; Sigma-Aldrich, St. Louis, MO, USA; 10 μM in PBS) and 10 μL DAPI. Following incubation (37 °C, 30 min, dark conditions), all specimens were analyzed with an epifluorescence microscope with 40× magnification. At least 300 spermatozoa were counted, and the cells exhibiting PNA-negativity were classified as acrosome-intact (%) [17].

### 2.5. Mitochondrial Membrane Potential (ΔΨm)

The mitochondrial membrane potential was evaluated with the JC-1 Mitochondrial Membrane Potential Assay kit (Cayman Chemical, Ann Arbor, MI, USA). The JC-1 dye (5.5′,6.6′-tetrachloro-1,1′,3,3′-tetraethylbenzimidazolylcarbocyanine iodide) was diluted in PBS shortly before the analysis, 5 μL of JC-1 working solution were mixed with 100 μL of each sample and incubated for 30 min at 37 °C. Subsequently the samples were centrifuged (5 min, 2100 RPM, 25 °C) and washed twice with a washing buffer provided by the kit. Lastly, the samples were transferred to a dark 96-chamber plate which was analyzed using the combined GloMax-Multi+ spectro-fluoro-luminometer (Promega, Madison, WI, USA). The resulting ΔΨm is expressed as the ratio of JC-1 complexes to JC-1 monomers (green/red ratio) [17].

### 2.6. Sperm DNA Damage

Sperm DNA fragmentation index (%) was quantified with the Halomax commercial kit (Halotech DNA, Madrid, Spain). Briefly, 20 μL of each specimen were mixed with low-melting point agarose. Ten μL of the mixture were transferred onto slides pre-coated with agarose, covered with coverslips, and stored at 4 °C for 5 min. Afterwards, the samples were treated with a lysis solution (5 min), distilled water (5 min), 70% and 100% ethanol (2 min each) and finally air-dried. Each slide was stained with SYBR Green (2 μg/mL) (Sigma-Aldrich, St. Louis, MO, USA) and Vectashield (Vector Laboratories, Burlingame, CA, USA) and at least 300 cells were evaluated under an epifluorescence microscope with a 40× magnification objective [17].

### 2.7. Reactive Oxygen Species (ROS) Generation

The degree of ROS production was assessed with luminol-based chemiluminescence. The tested specimens involved 10 μL 5 mM luminol (Sigma-Aldrich, St. Louis, MO, USA) and 400 μL sample. Negative controls consisted of 400 μL of each extender. Positive controls contained 400 μL of each extender and 50 μL hydrogen peroxide (H_2_O_2_; 30%; 8.8 M; Sigma-Aldrich, St. Louis, MO, USA). The resulting reaction was monitored in fifteen 1-min cycles on 48-well microplates using the Glomax Multi^+^ combined spectro-fluoro-luminometer (Promega, Madison, WI, USA). The extent of ROS generation is expressed in relative light units (RLU)/s/10^6^ sperm [18].

### 2.8. Bacteriological Analysis

For the description of the bacterial colonies and species in extended boar semen, 100 µL of each specimen were inoculated onto sterile blood, Gassner, MacConkey and Tryptic soy agar (MHB, Oxoid, Basingstoke, UK) under aerobic conditions (37 °C; 24–48 h). Colony-forming units (CFU/mL) were counted and purified by the four-way streak plate method [15].

### 2.9. Identification of Bacteria

The purified bacterial colonies were identified with the help of the MALDI-TOF Biotyper mass spectrometry (Brucker Daltonics, Bremen, Germany).

A small amount of each purified culture was mixed with 300 μL distilled water. Subsequently, 900 μL 99.8% ethanol (Centralchem, Bratislava, Slovakia) were added and the samples were centrifuged at 3200 RPM for 2 min at laboratory temperature. The resulting pellet was left to dry freely and subsequently resuspended in 30 μL 70% formic acid (Sigma-Aldrich, St. Louis, MO, USA) and the same amount of acetonitrile (Sigma-Aldrich, St. Louis, MO, USA). Following a second round of centrifugation (3500 RPM, laboratory temperature 2 min), 1 μL of the supernatant was placed on a 96-point MALDI identification plate and left to dry freely [15,18,19].

Acetonitrile, ultrapure water, and trifluoroacetic acid (Sigma-Aldrich, St. Louis, MO, USA) were mixed in a ratio of 20:19:1 to prepare the working solution of the MALDI matrix. A small amount of cinnamic acid powder (Sigma-Aldrich, St. Louis, MO, USA) was mixed with 250 μL of the MALDI solution and the resulting mixture was poured over the plate with the dried supernatant. Bacterial identification was performed with the Microflex LT instrument equipped with the flexControl software version 3.4. The spectra measured by mass spectrometry were compared with the MALDI Biotyper Bruker Taxonomy database (Bruker Daltonics, Bremen, Germany) [15,18,19].

### 2.10. Antibiotic Resistance Testing

Bacterial species isolated from extended boar semen were tested for antibiotic resistance. The antimicrobial susceptibility test was performed with the disc diffusion method against (10 µg per disc) tobramycin (TOB), imipenem (IMP), tigecyklin (TGC), ampicillin (AMP), chloramphenicol (C), tetracycline (TET), ciprofloxacin (CIP), meropenem (MEM) and MIC strips (0.25–4 mg/L) for *C. difficile* according to Kačániová et al. [20].

### 2.11. Statistical Analysis

The collected data were statistically evaluated with the GraphPad Prism program (version 8.4.3 for Mac; GraphPad Software, La Jolla, CA, USA). Descriptive statistical characteristics (mean, standard deviation) together with One-way ANOVA and Tukey multiple comparison test were selected for the analysis. The level of significance was set at *** *p* < 0.001; ** *p* < 0.01; * *p* < 0.05.

## 3. Results

Changes to the sperm motility following exposure to selected semen extenders are shown in Table 1. The assessment at 24 h and 48 h revealed that the decline of sperm motility was significantly slower in the experimental group 3 (samples extended with Andostar Plus supplemented with gentamicin, lincomycin and spectinomycin) in comparison with the control (*p* < 0.05). Following 72 h, a significantly higher motility was detected in case of the experimental groups 2 (*p* < 0.05) and 3 (*p* < 0.01) when compared to the control. Furthermore, the motility was significantly higher in the presence of Androstar Plus supplemented with gentamicin, lincomycin and spectinomycin (Exp 3) in comparison to the extender carrying gentamycin exclusively (Exp 1; *p* < 0.05).

Evaluation of spermatozoa with intact membranes was carried out using fluorescent microscopy and the CFDA probe. A significant protective effect of Androstar Plus with the combination of gentamicin, lincomycin and spectinomycin (Exp 3) on the sperm plasma membrane became evident after 24 h (*p* < 0.05), and this trend was maintained following 48 h (*p* < 0.01) and 72 h (*p* < 0.001) of sperm storage (Table 2). Similarly, semen storage in Androstar Plus with gentamicin, aminoglycosid and cephalosporin (Exp 2) led to a higher proportion of viable spermatozoa following 48 h and 72 h when compared to the control group (*p* < 0.01). While a higher percentage of viable spermatozoa was observed in the experimental group 1, no significant differences in comparison with the control were observed. No significant differences were observed among the experimental groups.

Fluorescent analysis carried out to assess the proportion of dead spermatozoa revealed no effect of the antibiotic supplements on the male gametes as the percentage of PI-positive spermatozoa in the experimental groups did not differ from the control samples (Table 3) throughout the experiment. However, a moderate decrease in the proportion of dead spermatozoa was observed in all experimental groups during all assessment times. Differences in the proportion of necrotic cells among the experimental groups remained insignificant.

Supplementation of antibiotics to the semen extender did not instantly affect the acrosome status of the spermatozoa, as the percentage of acrosome-intact cells was not statistically different among the control and experimental groups (Table 4). However, a lower percentage of acrosome-damaged spermatozoa was observed in the experimental group 3 in comparison to the control following 48 h (*p* < 0.05) and 72 h (*p* < 0.01). Furthermore, the fluorescent assessment at 72 h revealed that the proportion of acrosome-intact spermatozoa preserved in Androstar Plus with gentamicin, lincomycin and spectinomycin (Exp 3) was significantly higher in comparison to Androstar Plus containing only gentamicin (Exp 1; *p* < 0.05). A significantly higher percentage of spermatozoa with a preserved acrosome was also detected in the experimental group 2 in comparison to the control (*p* < 0.05) following 72 h of storage. Moreover, the proportion of spermatozoa with an intact acrosome was significantly higher in the presence of Androstar Plus supplemented with gentamicin, lincomycin and spectinomycin (Exp 3) when compared to the extender containing only gentamycin (Exp 1; *p* < 0.05).

The JC-1 assay at 24 h revealed a significantly higher mitochondrial membrane potential in case of boar spermatozoa stored in the presence of gentamicin, aminoglycosid and cephalosporin (Exp 2) in comparison with the control (*p* < 0.05). Furthermore, spermatozoa extended in Androstar with gentamicin, lincomycin and spectinomycin (Exp 3) exhibited a significantly higher mitochondrial activity (*p* < 0.05) when compared to the control (Table 5). Beneficial effects of both combinations of antibiotics on the mitochondrial metabolism were observed following 48 h as well (*p* < 0.05). At the end of the experiment, the highest mitochondrial activity was detected in the experimental group 3 (*p* < 0.001). A significantly higher mitochondrial membrane potential was also detected in case of the experimental group 2 (*p* < 0.001) as well as in the experimental group 1 (*p* < 0.05) when compared to the control. At the same time, no significant differences were recorded among the experimental groups.

Data collected from the chromatin dispersion test (Table 6) revealed DNA-stabilizing effects of the antibiotics, particularly in the case of the experimental group 3, which became evident after 24 h (*p* < 0.05) and remained significant throughout the entire experiment (*p* < 0.05). Similarly, a significantly lower proportion of cells with fragmented DNA was recorded in the presence of gentamicin, aminoglycosid and cephalosporin (Exp 2) in comparison to the antibiotic-free control (*p* < 0.05). The analysis showed that the selection of antibiotics in the experimental groups had no significant effects on the extent of sperm DNA damage.

To assess the potential of selected boar semen extenders to prevent potential ROS overproduction as a result of bacteriospermia, we used a luminometric approach using luminol as the probe, which has been extensively used to study the global ROS production by sperm in mammalian and avian semen [15,18,19] (Table 7). At 24 h the amount of ROS significantly decreased in the experimental group 3 in comparison with the control (*p* < 0.05). This ability to prevent ROS overproduction remained significant following 48 h in the case of the experimental groups 2 and 3 (*p* < 0.01). Significant ROS-quenching properties of all extenders containing antibiotics (*p* < 0.05 for Exp 1; *p* < 0.01 in case of Exp 2; *p* < 0.001 with respect to Exp 3) were detected following 72 h of storage. No significant differences were recorded among the experimental groups.

Using MALDI-TOF mass spectrometry, 12 genera, and 16 bacterial species were identified in boar ejaculates immediately following semen dilution (Table 8): *Proteus vulgaris* (*P. vulgaris*), *Escherichia coli* (*E. coli*), *Pseudomonas aeruginosa* (*P. aeruginosa*), *Pseudomonas putida* (*P. putida*), *Klebsiella pneumoniae* (*K. pneumoniae*), *Aerococcus viridans* (*A. viridans*), *Staphylococcus aureus* (*S. aureus*), *Staphylococcus chromogenes* (*S. chromogenes*), *Staphylococcus simulans* (*S. simulans*), *Clostridium difficile* (*C. difficile*), *Enterococcus hirae* (*E. hirae*), *Bacillus cereus* (*B. cereus*), *Bacillus licheniformis* (*B. licheniformis*), *Bacillus subtilis* (*B. subtilis*), *Acinetobacter iwoffii* (*A. iwoffii*), *Rothia nasimurium* (*R. nasimurium*) and *Corynebacterium* spp. A detailed description of the bacteriocenoses identified in each sample is listed in Table 8.

A significant decrease (*p* < 0.001) in the bacterial load in comparison to the control group was noted after 24 h in all experimental groups (Table 9). While all bacterial species remained present in the control group following 24 h of storage, *A. viridans*, *C. difficile*, *E. hirae*, *Corynebacterium* spp. and *B. subtilis* were identified in samples exposed to Androstar Plus supplemented with gentamycin. *C. difficile*, *E. hirae* and *B. subtilis* were detected in the presence of gentamicin, aminoglycosid and cephalosporin, *Corynebacterium* spp. was found in semen samples exposed to gentamicin, lincomycin and spectinomycin.

Following 48 h of semen storage, a slight increase of bacterial load was recorded in the control group, while a significantly reduced (*p* < 0.001) number of bacterial colonies was observed in all experimental groups. Bacterial profiles did not change significantly in comparison to the previous assessment time, except for the experimental group 2, antibiotics present in which were able to eliminate *C. difficile*.

The final assessment at 72 h of semen storage revealed a continuous time-dependent increase of bacterial colonies in the control group while the quantity of bacterial colonies in the experimental groups continuously decreased with time. As with previous assessment times, the quantity of bacterial colonies present in the experimental groups was significantly lower in comparison with the control (*p* < 0.001). With respect to the variability of bacterial species present in the samples, all bacteria present in the control group continued to be present throughout the experiment, while *E. hirae*, *Corynebacterium* spp. and *B. subtilis* continued to be identified in Androstar Plus containing gentamycin exclusively. The lowest quantity of bacteria as well as their variability was observed in the experimental groups 2 and 3, with *E. hirae* and *B. subtilis* being isolated from the experimental group 2, while only *Corynebacterium* spp. was retrieved from the experimental group 3 (Table 9).

All bacterial isolates retrieved from boar semen were tested for antimicrobial resistance (Table 10) against tobramycin, imipenem, tigecyklin, ampicillin, chloramphenicol, tetracykline, ciprofloxacin and meropenem. The resulting inhibition zones were assessed in accordance with the European Committee on Antimicrobial Susceptibility Testing (EUCAST) recommendations. All *A. iwoffii*, *E. coli*, *K. peumoniae*, *P. vulgaris*, *P. aeruginosa*, *P. putida*, *S. aureus* and *S. chromogenes* isolates (100%) were sensitive to tobramycin, while all *A. iwoffii*, *E. hirae*, *E. coli*, *K. pneumoniae*, *P. vulgaris*, *P. aeruginosa* and *P. putida* isolates (100%) were sensitive to imipenem. All *E. hirae*, *E. coli*, *K. pneumoniae* and *P. vulgaris* isolates (100%) were furthermore sensitive to ampicillin. While *C. difficile* exhibited sensitivity to tigecyklin (100%), all *E. hirae*, *S. aureus* (100%) as well as 50% of *S. simulans* and *S. chromogenes* isolates were resistant to the antibiotic. Furthermore, all *A. iwoffi*, *P. aeruginosa* and *P. putida* isolates (100%) were resistant to ciprofloxacin.

## 4. Discussion

Numerous etiologies are currently acknowledged as contributors to a decreased quality of semen used for reproductive technologies, out of which bacteriospermia has recently gained in interest. In order to prevent the loss of sperm structural integrity and functional activity, and to minimize the risks of disease transmission to females, readily available data on the bacterial profiles of raw or extended semen may be crucial for further semen handling and use in the insemination process [3,4,5,6,7,8].

In comparison to conventional microbiological tools for bacterial screening, MALDI-TOF MS has emerged as a time-effective and reliable analytical approach for the identification of bacterial profiles in animal semen [15,18,19,21,22]. The advantage of such method lies in an early identification of pathogens, even at the level of strains or serotypes, which can significantly shorten the time of isolation and eventual initiation of treatment [23]. At present, however, 16S rRNA sequencing remains the “gold standard” of bacterial identification, since this technique does not rely on whether or not bacteria present in a sample are culturable. As such, it must be noted that even using a molecular approach with mass spectrometry, a full bacterial profile of the samples may be currently obtained by amplicon sequencing exclusively [24].

Our results indicate that 76% semen samples used for the experiments were contaminated by a variety of bacterial species, some of which are well-known uropathogens (*E. coli*, *P. aeruginosa*, *A. viridans*, *S. aureus*, *C. difficile*). As opposed to Dalmutt et al. [25] who reported only a 43% bacterial positivity of boar semen samples, Bennemann et al. [26] observed that up to 86% of boar ejaculates were infested with at least two different bacteria, while almost all ejaculates (99%) tested positive for bacteriospermia in the report by Gazarewitz et al. [4]. Bresciani et al. [5] observed a 63% positivity of boar semen samples collected in Italy.

Most of the samples tested positive for *E. coli* and *Pseudomonas*, which is consistent with previous studies on boar semen. Similarly to Bresciani et al. [5] and Maroto Martín et al. [6] we also identified representatives of the *Proteus* (36%) and *Staphylococcus* (12%) genera, which confirms the general view that boar ejaculates are more prone to be infested by Gram-negative (G^−^) aerobic bacteria, however *Citrobacter*, *Streptococcus* and *Serratia* were absent in our samples. In our study, we found *Rothia*, *Acinetobacter* and *Corynebacterium* while, interestingly, various species belonging to the *Bacillus* genus were isolated frequently from our samples, agreeing with Gazarewitz et al. [4] who observed that this bacterium was the most frequently isolated genus from stored boar semen samples.

Microbial contamination of semen may arise from a systemic and/or reproductive infection or during ejaculation when the ejaculate may enter into contact with bacteria routinely colonizing the reproductive tract. Furthermore, environmental contamination may occur during semen collection and processing and be caused by contaminated materials, equipment, and chemicals, as well as inappropriate semen conditioning [27]. Moreover, it has been speculated that the technique and hygiene of semen collection and processing may significantly contribute to semen contamination by bacteria [28]. Since all the boars included in this study were healthy, fertile, and showed no signs of urogenital infection, we may speculate that the quantity as well as diversity of bacteria present in the samples could be associated with faults in semen collection and handling, as well as by differences between the animals in terms of the native characteristics of their ejaculates [4]. Furthermore, while such bacterial profiles would not have been a major concern under in vivo conditions, since only a short-term interaction between bacteria and spermatozoa occurs during the ejaculation process, this interplay may be prolonged during semen storage, and possibly lead to a “silent ejaculate infection” with detrimental effects on the sperm quality [29].

The presence of bacteria in boar ejaculates as seen worldwide has created the necessity to use extenders that can assure an efficient protection of spermatozoa against a possible bacterial growth in this potentially contaminated environment. Since currently available commercial extenders contain one or a combination of antibiotics to accommodate the European Directive 90/429/EEC [16], our goal was to assess their potential to effectively limit bacterial growth under conditions that are currently being recommended for the storage of diluted boar semen.

This study, in agreement with previous reports in the field of AI practices in swine production [5,6,11,12] reveals that the occurrence of bacteria in extended semen is common, and antibiotic supplementation exhibits a limited control of bacterial persistence and growth during semen storage.

Data collected from our experiments indicate that gentamycin was effective enough to eradicate G^-^ bacteria while the incidence of Gram-positive (G^+^) bacteria was not significantly affected. In fact, samples diluted in AndroStar Plus containing gentamycin exclusively, were positive for G^+^ bacteria only. These changes in the diversity of bacteria may result from the inherent properties of gentamicin, which is more effective against G^-^ bacteria but otherwise it has a rather limited spectrum of activity [11]. In this sense, we may agree with Gączarzewicz et al. [4], Bresciani et al. [5] and Maroto Martín [6] who have emphasized that a significant proportion of bacteria routinely found in boar ejaculates in Europe may be resistant to gentamicin [30]. In the meantime, a combination of gentamycin with other antibiotics exhibited a higher degree of protection against the growth of G^+^ bacteria, as revealed by a significantly decreased bacterial load as well as a lower diversity of the species present in the experimental samples. Nevertheless, it has been previously reported that *Enterococcus* and *Bacillus* species may present with a high level of resistance against aminoglycosides and cephalosporins [31,32,33], while *Corynebacterium* was reported to contain phenotypes of multidrug resistance [34,35]. These patterns of bacterial behavior towards antibiotics may be responsible for our observations of the persistence of a small group of bacteria even in the presence of a combination of different antibiotics. This hypothesis is further confirmed by previous studies reporting that several bacterial genera and species exhibit a certain degree of resistance to gentamycin and aminoglycosides, which are among the most common antibiotic supplements found in semen extenders [7,11,12,36], leading to a concerning assumption that although extenders suitable for boar semen comply with the currently valid legislation, none of them was able to effectively eradicate bacteria present in diluted semen samples.

It has been emphasized earlier in this paper that sperm damage as a result of bacteriospermia in boar ejaculates becomes notable only if contamination rates are greater than 1.00 log CFU/mL [7,37,38]., This understanding was achieved with a combination of gentamicin, aminoglycosid and cephalosporin or gentamicin, lincomycin and spectinomycin in our experiments. As postulated by Maroto-Marín [6] and Bussalleu et al. [39], AI performed with a semen sample contaminated particularly with *E. coli* above a threshold value of 3.5 × 10^3^ CFU/mL may lead to significant reduction in litter size, which has not been observed in the case of extenders containing antibiotics in this study. Furthermore, it has been reported that potentially detrimental effects on the sow’s reproductive system by bacteria originating from boar semen are relatively low since estrous sows have a low susceptibility to uterine inflammation [40]. Summarizing these arguments, AI using semen samples with a low bacterial load may result in normal fertility outcomes and female reproductive health [41].

In this study, a 3-day semen storage period resulted in a variable reduction of sperm structural integrity and functional activity, depending on the antibiotic supplement used in the extender and thus on the bacterial load and/or diversity. Accordingly, the absence of antibiotics in the semen extender or the presence of gentamycin exclusively were unable to control the persistence of G^+^ bacteria, which may subsequently adhere to the sperm surface [42,43,44] and cause sperm agglutination through the release of agglutination or immobilization molecules [43]. Furthermore, numerous bacteria may attach themselves to the acrosomal or flagellar structures, causing the sperm flagellum to tear off, knot, or break and thus ceasing to move effectively [42,44]. A decrease of the sperm viability observed in the presence of an ineffective control of bacterial growth may be associated with the secretion of bacterial endotoxins, lipopolysaccharide (LPS) or peptidoglycan fragments, which may disrupt the integrity of the sperm plasma membrane and activate intracellular receptors for apoptotic or necrotic cell death [45].

Cytotoxic and inflammatory processes caused by the presence and activity of bacteria in semen are often accompanied by ROS overproduction. The resulting oxidative stress may ultimately cause more profound damage to the sperm cell, which is associated with an increased peroxidation of lipids present in the sperm plasma membrane [15,19]. Subsequent oxidative chain reactions will lead to alterations in the membrane fluidity and permeability, and disintegration of the internal milieu of the sperm cell, which will be translated into the loss of vitality and activity [14,15,19,46]. Furthermore, bacterial contamination has been frequently associated with an elevated sperm DNA fragmentation index [15,19,47,48]. An increased percentage of spermatozoa with fragmented DNA in the presence of a higher bacterial load may be associated with oxidative insults to the DNA molecule stemming from ROS overproduction and an elevated oxidative pressure [14,47]. This hypothesis has been also previously supported by positive associations between the extent of sperm DNA damage with the amounts of ROS and bacterial load. Moreover, as suggested by Ďuračka et al. [15] and Lenický et al. [19], the release of bacterial endotoxins triggers apoptosis or necrosis, which are accompanied by the loss of DNA stability [48].

Finally, it is of relevance to pay attention to the effects of antibiotics on the sperm structure and function. While it has been demonstrated that antibiotic supplementation to semen extenders is beneficial by inhibiting the bacterial growth in ejaculates during their storage, their direct impact on the structures crucial for the sperm cell to reach the egg and accomplish a successful fertilization are yet to be studied. While most previous reports [16,49] claim that currently available antibiotic supplements do not exhibit a negative effect on the sperm survival, more understanding on complex and perhaps unexpected effects on relevant production aspects such as fertility, prolificacy, fecundity, or sex-ratio are still necessary.

## 5. Conclusions

In this study, 76% of boar ejaculates were positive for the presence of bacteria. with a predominance of *E. coli* and *Pseudomonas*. It is highlighted that gentamycin alone was not able to prevent the occurrence of G^+^ bacteria in extended semen samples which was accompanied by a lower sperm quality over 72 h of semen storage. The lowest bacterial load and variability followed by a significant preservation of the sperm structural integrity and functional activity were recorded in the presence of gentamycin, lincomycin and spectinomycin. Finally, it may be concluded that the quality of extended boar semen depends on the antibacterial supplement added to the diluent. Furthermore, the selection of appropriate antibiotics for semen processing must come hand in hand with optimal and thorough hygiene measures during semen collection, dilution, and storage. Also, frequent, and periodic bacteriological screening of boar semen should become routine in the swine industry in order to avoid the use of low-quality ejaculates for artificial insemination.

## Figures and Tables

**Table 1:** Motility of boar spermatozoa (%) exposed to selected semen extenders during different time intervals.

Time of Analysis	Ctrl	Exp 1	Exp 2	Exp 3
0 h	78.54 ± 7.22			
24 h	66.22 ± 5.58	72.03 ± 8.11	75.56 ± 8.99	77.79 ± 9.03 *^Ctrl^
48 h	54.79 ± 4.99	62.91 ± 8.10	67.57 ± 9.03	71.33 ± 7.44 *^Ctrl^
72 h	45.25 ± 3.68	56.19 ± 7.07	61.28 ± 4.98 *^Ctrl^	67.22 ± 5.87 **^Ctrl;^ *^Exp^ ^1^

** *p* < 0.01; * *p* < 0.05. ^Ctrl^–in comparison with the control; ^Exp 1^–in comparison with the Experimental group 1. Ctrl–Androstar Plus without antibiotics; Exp 1–Androstar Plus with gentamycin; Exp 2–Androstar Plus with gentamicin, aminoglycosid and cephalosporin; Exp 3–Androstar Plus with gentamicin, lincomycin and spectinomycin. *n* = 30.

**Table 2:** Viability of boar spermatozoa (%) exposed to selected semen extenders during different time intervals.

Time of Analysis	Ctrl	Exp 1	Exp 2	Exp 3
0 h	87.89 ± 7.36			
24 h	75.88 ± 6.55	78.91 ± 8.07	81.51 ± 7.21	84.67 ± 6.58 *^Ctrl^
48 h	59.14 ± 7.52	69.25 ± 7.57	72.17 ± 6.55 *^Ctrl^	75.22 ± 6.45 **^Ctrl^
72 h	46.78 ± 5.28	57.02 ± 6.35	62.14 ± 7.00 **^Ctrl^	68.09 ± 5.94 ***^Ctrl^

*** *p* < 0.001; ** *p* < 0.01; * *p* < 0.05. ^Ctrl^–in comparison with the control. Ctrl–Androstar Plus without antibiotics; Exp 1–Androstar Plus with gentamycin; Exp 2–Androstar Plus with gentamicin, aminoglycosid and cephalosporin; Exp 3–Androstar Plus with gentamicin, lincomycin and spectinomycin. *n* = 30.

**Table 3:** Occurrence of dead boar spermatozoa (%) following exposure to selected semen extenders during different time intervals.

Time of Analysis	Ctrl	Exp 1	Exp 2	Exp 3
0 h	8.00 ± 1.12			
24 h	10.22 ± 3.88	8.99 ± 1.08	8.54 ± 1.13	8.12 ± 0.87
48 h	13.09 ± 3.87	9.99 ± 1.00	8.99 ± 1.12	8.69 ± 1.03
72 h	15.88 ± 2.76	11.57 ± 1.07	10.33 ± 2.71	9.62 ± 0.98

Ctrl–Androstar Plus without antibiotics; Exp 1–Androstar Plus with gentamycin; Exp 2–Androstar Plus with gentamicin, aminoglycosid and cephalosporin; Exp 3–Androstar Plus with gentamicin, lincomycin and spectinomycin. *n* = 30.

**Table 4:** Acrosome integrity of boar spermatozoa (%) exposed to selected semen extenders during different time intervals.

Time of Analysis	Ctrl	Exp 1	Exp 2	Exp 3
0 h	91.06 ± 5.47			
24 h	80.34 ± 4.41	82.18 ± 6.25	84.26 ± 5.13	87.20 ± 4.53
48 h	72.55 ± 4.78	76.05 ± 5.03	80.80 ± 4.35	83.85 ± 6.06 *^Ctrl^
72 h	57.36 ± 3.12	61.09 ± 7.05	68.09 ± 4.75 *^Ctrl^	74.04 ± 6.25 **^Ctrl;^ *^Exp^ ^1^

** *p* < 0.01; * *p* < 0.05. ^Ctrl^–in comparison with the control; ^Exp 1^–in comparison with the Experimental group 1. Ctrl–Androstar Plus without antibiotics; Exp 1–Androstar Plus with gentamycin; Exp 2–Androstar Plus with gentamicin, aminoglycosid and cephalosporin; Exp 3–Androstar Plus with gentamicin, lincomycin and spectinomycin. *n* = 30.

**Table 5:** Mitochondrial membrane potential of boar spermatozoa (JC-1 units) exposed to selected semen extenders during different time intervals.

Time of Analysis	Ctrl	Exp 1	Exp 2	Exp 3
0 h	0.88 ± 0.09			
24 h	0.77 ± 0.09	0.80 ± 0.04	0.82 ± 0.05 *^Ctrl^	0.86 ± 0.07 *^Ctrl^
48 h	0.61 ± 0.05	0.75 ± 0.03	0.79 ± 0.06 *^Ctrl^	0.83 ± 0.03 *^Ctrl^
72 h	0.47 ± 0.05	0.63 ± 0.06 *^Ctrl^	0.68 ± 0.07 ***^Ctrl^	0.72 ± 0.06 ***^Ctrl^

*** *p* < 0.001; * *p* < 0.05. ^Ctrl^–in comparison with the control. Ctrl–Androstar Plus without antibiotics; Exp 1–Androstar Plus with gentamycin; Exp 2–Androstar Plus with gentamicin, aminoglycosid and cephalosporin; Exp 3–Androstar Plus with gentamicin, lincomycin and spectinomycin. *n* = 30.

**Table 6:** DNA fragmentation index of boar spermatozoa (%) exposed to selected semen extenders during different time intervals.

Time of Analysis	Ctrl	Exp 1	Exp 2	Exp 3
0 h	8.54 ± 0.98			
24 h	13.22 ± 1.03	12.58 ± 1.27	12.14 ± 1.08	9.99 ± 0.94 *^Ctrl^
48 h	18.55 ± 1.88	16.02 ± 1.77	14.06 ± 1.15	12.15 ± 1.04 *^Ctrl^
72 h	24.21 ± 1.99	20.00 ± 2.12	17.50 ± 2.07 *^Ctrl^	16.87 ± 2.01 *^Ctrl^

* *p* < 0.05. ^Ctrl^–in comparison with the control. Ctrl–Androstar Plus without antibiotics; Exp 1–Androstar Plus with gentamycin; Exp 2–Androstar Plus with gentamicin, aminoglycosid and cephalosporin; Exp 3–Androstar Plus with gentamicin, lincomycin and spectinomycin. *n* = 30.

**Table 7:** Reactive oxygen species production by boar spermatozoa (RLU/s/10^6^ cells) exposed to selected semen extenders during different time intervals.

Time of Analysis	Ctrl	Exp 1	Exp 2	Exp 3
0 h	7.07 ± 0.87			
24 h	16.58 ± 1.99	14.07 ± 2.09	11.88 ± 1.79	9.55 ± 1.11 *^Ctrl^
48 h	28.45 ± 3.60	21.74 ± 2.05	18.74 ± 2.05 **^Ctrl^	16.07 ± 2.66 **^Ctrl^
72 h	37.25 ± 4.08	27.14 ± 2.14 *^Ctrl^	25.33 ± 3.02 **^Ctrl^	21.01 ± 2.77 ***^Ctrl^

*** *p* < 0.001; ** *p* < 0.01; * *p* < 0.05. ^Ctrl^–in comparison with the control. Ctrl–Androstar Plus without antibiotics; Exp 1–Androstar Plus with gentamycin; Exp 2–Androstar Plus with gentamicin, aminoglycosid and cephalosporin; Exp 3–Androstar Plus with gentamicin, lincomycin and spectinomycin. *n* = 30.

**Table 8:** Bacterial profiles of boar semen samples used in the study.

Sample	Bacterial Isolates	Bacterial Colonies (log CFU/mL)
1	*C. difficile*, *E. hirae*, *P. vulgaris*, *P. aeuoginosa*, *P. putida*, *S. chromogenes*, *S. simulans*	5.44
2	ND	0.00
3	ND	0.00
4	*E. coli*, *P. aeuoginosa*, *R. nasimurium*, *S. aureus*	4.84
5	*A. viridans*, *Corynebacterium* sp., *E. hirae*, *E. coli*, *P. aeuoginosa*, *P. putida*	5.86
6	*B. cereus*, *B. licheniformis*, *B. subtilis*, *E. coli*, *K. pneumoniae*, *P. aeuoginosa*, *S. chromogenes*	6.98
7	*C. difficile*, *E. coli*, *P. aeuoginosa*	4.73
8	*E. coli*, *P. vulgaris*, *R. nasimurium*	8.02
9	ND	0.00
10	*A. iwoffii*, *S. aureus*	7.08
11	*E. coli*, *R. nasimurium*	4.52
12	ND	0.00
13	*A. iwoffii*, *B. licheniformis*, *E. coli*, *P. aeuoginosa*	6.19
14	*E. coli*, *P. vulgaris*, *P. aeuoginosa*	7.08
15	*E. coli*, *P. aeuoginosa*, *S. simulans*	6.28
16	*Bacillus subtilis*, *Corynebacterium* sp., *C. difficile*, *E. hirae*, *E. coli*, *P. aeuoginosa*	4.61
17	*C. difficile*, *E. hirae*, *E. coli*	6.05
18	*P. aeuoginosa*	7.11
19	ND	0.00
20	*A. viridans*, *B. subtilis*, *B. licheniformis*, *K. pneumoniae*, *P. putida*	4.58
21	*E. coli*, *P. aeuoginosa*, *S. chromogenes*, *R. nasimurium*	7.28
22	*C. difficile*, *E. coli*, *P. aeuoginosa*	4.37
23	*E. coli*, *P. aeuoginosa*	6.12
24	ND	0.00
25	*B. cereus*, *B. licheniformis*, *B. subtilis*, *P. aeuoginosa*, *S. chromogenes*, *S. simulans*	4.27
26	*E. coli*, *P. vulgaris*, *P. aeuoginosa*	5.55
27	*E. coli*, *P. aeuoginosa*	4.71
28	*E. hirae*, *E. coli*, *P. aeuoginosa*, *S. chromogenes*	5.88
29	*E. coli*, *P. vulgaris*	6.37
30	ND	0.00

**Table 9:** Bacteria recovered (% sample positivity) from extended boar semen and identified by MALDI-TOF MS Biotyper.

	Bacterial Isolates	Bacterial Colonies (log CFU/mL)
0 h		
Ctrl	*E. coli* (60%), *P. aeruginosa* (55%), *P. vulgaris* (17%), *C. difficile* (17%), *E. hirae* (13%), *S. chromogenes* (13%), *B. subtilis* (13%), *B. licheniformis* (13%), *R. nasimurium* (13%), *S. simulans* (13%), *P. putida* (6%), *K. pneumoniae* (6%), *A. viridans* (6%), *S. aureus* (6%), *B. cereus* (6%), *A. iwoffii* (6%), *Corynebacterium* spp. (6%)	4.50 ± 2.69
24 h		
Ctrl	*E. coli* (60%), *P. aeruginosa* (55%), *P. vulgaris* (17%), *C. difficile* (17%), *E. hirae* (13%), *S. chromogenes* (13%), *B. subtilis* (13%), *B. licheniformis* (13%), *R. nasimurium* (13%), *S. simulans* (13%), *P. putida* (7%), *K. pneumoniae* (7%), *A. viridans* (7%), *S. aureus* (7%), *B. cereus* (7%), *A. iwoffii* (7%), *Corynebacterium* spp. (7%)	4.89 ± 2.03
Exp 1	*A. viridans* (7%), *C. difficile* (13%), *E. hirae* (13%), *Corynebacterium* spp. (7%), *B. subtilis* (7%)	1.28 ± 0.32 ***^Ctrl^
Exp 2	*C. difficile* (7%), *E. hirae* (7%), *B. subtilis* (7%)	1.00 ± 0.18 ***^Ctrl^
Exp3	*Corynebacterium* spp. (7%)	0.96 ± 0.47 ***^Ctrl^
48 h		
Ctrl	*E. coli* (60%), *P. aeruginosa* (55%), *P. vulgaris* (17%), *C. difficile* (17%), *E. hirae* (13%), *S. chromogenes* (13%), *B. subtilis* (13%), *B. licheniformis* (13%), *R. nasimurium* (13%), *S. simulans* (13%), *P. putida* (7%), *K. pneumoniae* (7%), *A. viridans* (7%), *S. aureus* (7%), *B. cereus* (7%), *A. iwoffii* (7%), *Corynebacterium* spp. (7%)	5.12 ± 1.98
Exp 1	*A. viridans* (7%), *C. difficile* (7%), *E. hirae* (13%), *Corynebacterium* spp. (7%), *B. subtilis* (7%)	1.17 ± 0.57 ***^Ctrl^
Exp 2	*E. hirae* (7%), *B. subtilis* (7%)	0.94 ± 0.25 ***^Ctrl^
Exp 3	*Corynebacterium* spp. (7%)	0.80 ± 0.37 ***^Ctrl^
72 h		
Ctrl	*E. coli* (60%), *P. aeruginosa* (55%), *P. vulgaris* (17%), *C. difficile* (17%), *E. hirae* (13%), *S. chromogenes* (13%), *B. subtilis* (13%), *B. licheniformis* (13%), *R. nasimurium* (13%), *S. simulans* (13%), *P. putida* (7%), *K. pneumoniae* (7%), *A. viridans* (7%), *S. aureus* (7%), *B. cereus* (7%), *A. iwoffii* (7%), *Corynebacterium* spp. (7%)	5.24 ± 1.97
Exp 1	*A. viridans* (7%), *C. difficile* (7%), *E. hirae* (7%), *Corynebacterium* spp. (7%), *B. subtilis* (7%)	1.09 ± 0.33 ***^Ctrl^
Exp 2	*E. hirae* (7%), *B. subtilis* (7%)	0.52 ± 0.45 ***^Ctrl^
Exp 3	*Corynebacterium* spp. (7%)	0.44 ± 0.22 ***^Ctrl^

*** *p* < 0.001. ^Ctrl^–in comparison with the control. Ctrl–Androstar Plus without antibiotics; Exp 1–Androstar Plus with gentamycin; Exp 2–Androstar Plus with gentamicin, aminoglycosid and cephalosporin; Exp 3–Androstar Plus with gentamicin, lincomycin and spectinomycin. *n* = 30.

**Table 10:** Resistance profiles of bacteria recovered from extended boar semen.

Microorganisms		TOB	IMP	TGC	AMP	C	TET	CIP	MEM
*Acinetobacter iwoffii*	S	100%	100%	ND	ND	ND	ND	0%	ND
	I	0%	0%	ND	ND	ND	ND	0%	ND
	R	0%	0%	ND	ND	ND	ND	100%	ND
*Aerococcus viridans*	S	ND	ND	ND	ND	ND	ND	ND	ND
	I	ND	ND	ND	ND	ND	ND	ND	ND
	R	ND	ND	ND	ND	ND	ND	ND	ND
*Bacillus cereus*	S	ND	ND	ND	ND	ND	ND	ND	ND
	I	ND	ND	ND	ND	ND	ND	ND	ND
	R	ND	ND	ND	ND	ND	ND	ND	ND
*Bacillus licheniformis*	S	ND	ND	ND	ND	ND	ND	ND	ND
	I	ND	ND	ND	ND	ND	ND	ND	ND
	R	ND	ND	ND	ND	ND	ND	ND	ND
*Bacillus subtilis*	S	ND	ND	ND	ND	ND	ND	ND	ND
	I	ND	ND	ND	ND	ND	ND	ND	ND
	R	ND	ND	ND	ND	ND	ND	ND	ND
*Clostridium difficile* *	S	ND	ND	100%	ND	ND	ND	ND	100%
	I	ND	ND	0%	ND	ND	ND	ND	0%
	R	ND	ND	0%	ND	ND	ND	ND	0%
*Corynebacterium* spp.	S	ND	ND	ND	ND	ND	100%	100%	ND
	I	ND	ND	ND	ND	ND	0%	0%	ND
	R	ND	ND	ND	ND	ND	0%	0%	ND
*Enterococcus hirae*	S	ND	100%	0%	100%	ND	ND	ND	ND
	I	ND	0%	0%	0%	ND	ND	ND	ND
	R	ND	0%	100%	0%	ND	ND	ND	ND
*Escherichia coli*	S	100%	100%	ND	100%	ND	ND	ND	ND
	I	0%	0%	ND	0%	ND	ND	ND	ND
	R	0%	0%	ND	0%	ND	ND	ND	ND
*Klebsiella pneumoniae*	S	100%	100%	ND	100%	ND	ND	ND	ND
	I	0%	0%	ND	0%	ND	ND	ND	ND
	R	0%	0%	ND	0%	ND	ND	ND	ND
*Proteus vulgaris*	S	100%	100%	ND	100%	ND	ND	ND	ND
	I	0%	0%	ND	0%	ND	ND	ND	ND
	R	0%	0%	ND	0%	ND	ND	ND	ND
*Pseudomonas aeroginosa*	S	100%	100%	ND	ND	ND	ND	0%	ND
	I	0%	0%	ND	ND	ND	ND	0%	ND
	R	0%	0%	ND	ND	ND	ND	100%	ND
*Pseudomonas putida*	S	100%	100%	ND	ND	ND	ND	0%	ND
	I	0%	0%	ND	ND	ND	ND	0%	ND
	R	0%	0%	ND	ND	ND	ND	100%	ND
*Rothia nasimurium*	S	ND	ND	ND	ND	ND	ND	ND	ND
	I	ND	ND	ND	ND	ND	ND	ND	ND
	R	ND	ND	ND	ND	ND	ND	ND	ND
*Staphylococcus aureus*	S	100%	ND	0%	ND	100%	ND	ND	ND
	I	0%	ND	0%	ND	0%	ND	ND	ND
	R	0%	ND	100%	ND	0%	ND	ND	ND
*Staphylococcus chromogenes*	S	50%	ND	50%	ND	100%	ND	ND	ND
	I	0%	ND	0%	ND	0%	ND	ND	ND
	R	50%	ND	50%	ND	0%	ND	ND	ND
*Staphylococcus simulans*	S	100%	ND	50%	ND	100%	ND	ND	ND
	I	0%	ND	0%	ND	0%	ND	ND	ND
	R	0%	ND	50%	ND	0%	ND	ND	ND

TOB–Tobramycin, IMP–Imipenem, TGC–Tigecyklin, AMP–Ampicillin, C–Chloramphenicol, TET–Tetracykline, CIP–Ciprofloxacin, MEM–Meropenem, ND–not defined, S–sensitive, I–intermediate, R–resistant, *–MIC strips for *Clostridium difficile*.

## Data Availability

The data presented in this study are available on request from the corresponding author.
